# Incremental utility of expanded mutation panel when used in combination with microRNA classification in indeterminate thyroid nodules

**DOI:** 10.1002/dc.24328

**Published:** 2019-11-01

**Authors:** Sara Jackson, Gyanendra Kumar, Anna B. Banizs, Nicole Toney, Jan F. Silverman, Christina M. Narick, Sydney D. Finkelstein

**Affiliations:** ^1^ Division of Research & Development, Interpace Diagnostics, Inc. Pittsburgh Pennsylvania; ^2^ Division of Research & Development, Interpace Diagnostics, Inc. New Haven Connecticut; ^3^ Department of Pathology Yale School of Medicine New Haven Connecticut; ^4^ Department of Pathology Allegheny General Hospital Pittsburgh Pennsylvania; ^5^ Division of Pathology, Interpace Diagnostics, Inc. Pittsburgh Pennsylvania

**Keywords:** classification, microRNA, malignancy, mutation analysis, thyroid nodules

## Abstract

**INTRODUCTION:**

Focused and expanded mutation panels were assessed for the incremental utility of using an expanded panel in combination with microRNA risk classification.

**METHODS:**

Molecular results were reviewed for patients who underwent either a focused mutation panel (ThyGenX®) or an expanded mutation panel (ThyGeNEXT®) for strong and weak oncogenic driver mutations and fusions. microRNA results (ThyraMIR®) predictive of malignancy, including strong positive results highly specific for malignancy, were examined.

**RESULTS:**

Results of 12 993 consecutive patients were reviewed (focused panel = 8619, expanded panel = 4374). The expanded panel increased detection of strong drivers by 8% (*P* < .001), with *BRAFV600E* and *TERT* promoters being the most common. Strong drivers were highly correlated with positive microRNA results of which 90% were strongly positive. The expanded panel increased detection of coexisting drivers by 4% (*P* < .001), with *TERT* being the most common partner often paired with *RAS*. It increased the detection of weak drivers, with *RAS* and *GNAS* being the most common. 49% of nodules with weak drivers had positive microRNA results of which 33% were strongly positive. The expanded panel also decreased the number of nodules lacking mutations and fusions by 15% (*P* < .001), with 8% of nodules having positive microRNA results of which 22% were strongly positive.

**CONCLUSIONS:**

Using expanded mutation panels that include less common mutations and fusions can offer increased utility when used in combination with microRNA classification, which helps to identify high risk of malignancy in the cases where risk is otherwise uncertain due to the presence of only weak drivers or the absence of all drivers.

## 1 INTRODUCTION

Standard of care for the diagnosis of thyroid nodule malignancy includes ultrasound imaging followed by cytopathology review of fine‐needle aspirations (FNA). However, up to 30% of nodule FNAs will result in a cytology considered indeterminate for malignancy.^1^, ^2^, ^3^, ^4^ These cytology results can include Bethesda Diagnostic Category III (B‐III), which represents atypical cells with undetermined significance or follicular lesion of undetermined significance (AUS/FLUS), or Bethesda Diagnostic Category IV (B‐IV), which is indicative of follicular neoplasm or is suspicious for follicular neoplasm (FN/SFN). Malignancy risk can range from 12% to 33% in these indeterminate nodules.^5^ Without additional testing, diagnostic lobectomy is often required for definitive diagnosis, concluding most frequently in benign disease in the form of nodular hyperplasia, follicular adenoma, or related noncancerous processes for which tissue resection may have been unnecessary. These unnecessary surgeries result in significant healthcare costs and can negatively impact patient's quality of life.^6^

When faced with an indeterminate cytology diagnosis, molecular testing is often used in routine clinical practice to further assess the risk of malignancy. There are a variety of commercially available tests on the market, each of which takes a different approach to assessing malignancy risk. One approach includes the use of mutation and messenger RNA fusion biomarker panels (ie, mutation panels) to identify oncogenic driver changes that may be present. More recently, these mutation panels have been expanded to include additional markers for mutations and fusions, with the goal of better predicting increased risk for malignancy and limiting diagnostic dilemmas encountered when nodules lack detectable oncogenic changes, where 5% to 25% risk of cancer exists.^7^, ^8^, ^9^ However, inclusion of additional mutations and fusions that is not highly specific for malignancy may result in panels with lower positive predictive value (PPV).

Certain oncogenic drivers are strongly predictive of malignancy and aggressive thyroid cancer and as such can be considered “strong drivers.” These include *BRAFV600E*, *TERT* promoter mutations (C228T and C250T), and *RET* mutations as well as *BRAF* and *RET*‐related messenger RNA fusion transcripts. *BRAFV600E* and *RET* mutations and *RET* fusions have high PPV for malignancy, can be found in aggressive thyroid cancers, and are rarely if ever found in benign adenomas or hyperplastic nodules.^10^, ^11^, ^12^ *BRAF*‐related fusion transcripts have also been associated with *BRAFV600E*‐like properties, although they are more commonly found in pediatric thyroid cancer populations^11^, ^13^, ^14^, ^15^ *TERT* promoter mutations have been strongly correlated to persistent disease, aggressive forms of cancer, distant metastasis, and mortality.^16^, ^17^, ^18^, ^19^, ^20^, ^21^

Other oncogenic drivers can present a challenge to guiding patient management when they alone are used to assess malignancy risk. These drivers have been more weakly associated with malignancy in thyroid nodules and as such can be considered “weak drivers.” *RAS* mutations are the most common to indeterminate nodules and have been found in both benign and malignant nodules, presenting an uncertain PPV ranging from 15% to 70%.^8^, ^22^, ^23^, ^24^, ^25^ Other mutations and fusions can occur at a much lower frequency, making their PPV difficult to study and consequently not well understood. Rare *BRAF* mutations, excluding *BRAFV600E*, have *RAS*‐like properties rather than *BRAFV600E*‐like properties, and have been found in both benign and malignant thyroid nodules.^15^, ^26^, ^27^ Although *PIK3CA* and *PTEN* mutations have been reported in follicular thyroid cancer, poorly differentiated thyroid cancers, and anaplastic thyroid cancer,^28^, ^29^, ^30^ they can also be found in benign thyroid adenomas, as can *GNAS* and *ALK* mutations and *THADA‐* and *PPARG*‐related fusions.^28^, ^31^, ^32^, ^33^, ^34^, ^35^, ^36^, ^37^, ^38^, ^39^, ^40^, ^41^, ^42^ Furthermore, *PPARG‐* and *THADA*‐related fusion transcripts can have *RAS*‐like properties,^15^ while other fusions such as those related to *NTRK* and *ALK* have properties that are neither *RAS*‐like nor *BRAFV600E*‐like.^15^ Although the predictive value for malignancy of these oncogenic changes is not well understood when found individually, it is well established that coexistence of many of these oncogenic changes along with other oncogenic drivers is generally associated with aggressive forms of thyroid cancer and poor prognosis.^28^, ^29^, ^33^, ^43^, ^44^, ^45^

microRNA risk classifier testing has been used to help resolve diagnostic dilemmas encountered with the use of mutation panels alone. The microRNA classifier further assesses malignancy risk in nodules when mutation panels result in no oncogenic changes detected or in identification of oncogenic changes that have lower or less certain positive predictive value for malignancy. microRNAs reflect the output results of signaling pathways in a dynamic fashion providing valuable information of the behavior of the cells on the neoplastic spectrum. In contrast to mutations and fusions that occur at the intracellular level, microRNAs are uniquely designed to travel from one cell to another, regulating intercellular communication across multiple pathways, which places them central to understanding the transition from pre‐cancer states through malignant transformation and spread of cancers.^46^, ^47^, ^48^, ^49^, ^50^ A multiplatform approach using a combination of a mutation panel and a microRNA risk classifier has been shown to effectively “rule‐in” and “rule‐out” high risk of malignancy with results being predictive of surgical treatment decisions that are appropriately aligned with cancer risk.^6^, ^51^, ^52^ Furthermore, microRNA risk classification has been shown to help to reclassify cancer risk in both the absence of mutational change and the presence of mutations that have lower positive predictive values for malignancy, with strong positive microRNA classifier results offering extremely high specificity for malignancy.^8^

We aimed to better understand the incremental utility in using expanded mutation panels and how microRNA classifier testing can provide additional diagnostic information to expanded panel test results. We examined the frequency of strong and weak driver changes or the lack thereof in patients who underwent either focused mutation panel testing for more commonly tested strong and weak drivers or more robust, expanded mutation panel testing for additional strong and weak drivers. The latter cohort was also evaluated for microRNA risk classifications to determine how often positive and strong positive microRNA classifier results can elevate the risk of malignancy in patients who have undergone expanded panel testing.

## 2 MATERIALS AND METHODS

### 2.1 Patient cohorts

Molecular results of two cohorts of consecutive patients who had FNAs of thyroid nodules that underwent clinically prescribed focused mutation panel analysis (commercially known as ThyGenX®) from January 2017 to June 2018 or expanded mutation panel analysis (commercially known as ThyGeNEXT®) from June 2018 to November 2018 were examined. microRNA classifier test results (commercially nown as ThyraMIR®) were also examined to better understand the ability of those results to risk stratify patients who underwent more robust expanded panel testing. All patients in this study were reported as having Bethesda Diagnostic categories III or IV cytology results, or did not have a Bethesda Diagnostic category available in the data set examined. FNA specimens and corresponding cytology results were those from independent pathology practices, community hospital pathology departments, large metropolitan medical centers, and tertiary care academic centers in the United States and Canada. All molecular and cytology data were held in a secure central database as part of standard clinical practice. Use of de‐identified molecular and cytology data from the secure database was IRB approved (Quorum Review#: 31963) for use in this study. Informed consent was waived by the IRB due to minimal risk.

### 2.2 Molecular analysis

Molecular testing was prescribed by physicians as part of standard of care and performed at Interpace Diagnostics (Pittsburgh, Pennsylvania; New Haven, Connecticut) according to standard clinical practices. Molecular testing was performed on cytology smear slides or a separate FNA needle pass placed into RNA Retain (Assuragen) fixative solution depending on that prescribed by the physician.

For focused (ThyGenX®) and expanded (ThyGeNEXT®) mutation panel analyses, targeted next‐generation sequencing (NGS) (MiSeq, Illumina) was used to detect messenger RNA (mRNA) fusion transcripts and DNA mutation variants listed in Table 1 using polymerase chain reaction (PCR) to amplify the regions of interest prior to sequencing. A sequencing read depth of 1 000 was required for variant calls. Specimens were required to contain at least 3% of *BRAFV600E* for a positive variant call, 10% of *GNAS*, or 5% of the other individual DNA variants in the panel. For a positive mRNA fusion transcript call, specimens were required to contain at least 5% of an individual mRNA fusion transcript. Mutations and fusions common to both the focused and expanded mutation panels and those unique to the expanded panel are listed in Table 1. Mutations and fusions were categorized as strongly associated with thyroid malignancy and aggressive thyroid cancer (ie, strong drivers) or weakly associated (ie, weak drivers) based on published evidence described in the Introduction. Mutations and fusions in each of these categories for the focused and expanded panels are listed in Table 1 (bold vs regular font).

**Table 1:** Mutations and messenger RNA fusion transcripts included in both the focused and the expanded panels (gray cells) with additional mutations and fusions unique to the expanded panel indicated (white cells) [Color table can be viewed at http://wileyonlinelibrary.com]

DNA mutation markers	Messenger RNA fusion transcripts		
***BRAF V600E***	*PPARG_1/PAX8*	***RET/CCDC6***	***BRAF/AGK***
BRAF X*	*PPARG_2/PAX8*	***RET_4/NCOA4***	***BRAF/AKAP9***
*HRAS*	*PPARG_3/PAX8*	***RET_3/NCOA4***	***BRAF/SPTLC2***
*KRAS*	*PPARG_4/PAX8*	***RET_3d/NCOA4***	
*NRAS*	*PPARG/ CREB3L2*	***RET_5/GOLGA5***	*ALK/STRN*
*PIK3CA*		***RET/ELKS***	*ALK/EML4*
*ALK*	*NTRK2/TERT*	***RET/TRIM24***	
***RET***	*NTRK1/TPM3*	***RET/TRIM33***	*THADA/TRA2A*
***TERT* promoter**	*NTRK1/TFG*	***RET_8/KTN1***	*THADA28/LOC389473*
*GNAS*	*NTRK1‐1/TPR*	***RET_11/PCM1***	*THADA29/LOC389473*
*PTEN*	*NTRK1‐2/TPR*	***RET_9/RFG9***	*THADA31/IGF2BP3‐2*
	*NTRK3‐1/ETV6*	***RET/TRIM27***	*THADA30/IGF2BP3‐3*
	*NTRK3‐2/ETV6*	***RET/HOOK3***	
	*NTRK3/SLC12A6*	***RET/PRKARIA***	

*Notes*: Mutations and fusions were categorized as being strongly associated with malignancy and aggressive cancer (ie, strong drivers, bold font) or more weakly associated (ie, weak drivers, regular font) as described in the Introduction. BRAF X* indicates *BRAF* mutation other than *BRAFV600E*.

microRNA classification (ThyraMIR®) was based on a clinically validated panel of 10 specific microRNAs performed by quantitative real‐time PCR (QuantStudio) to evaluate microRNA expression levels in relation to each other.^51^, ^53^, ^54^ The panel of microRNAs included miR‐29b‐1‐5p, miR‐31‐5p, miR‐138‐1‐3p, miR‐139‐5p, miR‐146b‐5p, miR‐155, miR‐204‐5p, miR‐222‐3p, miR‐375, and miR‐551b‐3p. The microRNA classifier score was based on the relative overexpression or underexpression of the 10 microRNAs, resulting in a numerical value lying across a continuum from 0 to 1.0 as previously described.^55^ A value of 0 represented the most confident prediction of benignancy. A value of 1.0 represented the most confident prediction of malignancy. microRNA results were classified as negative or positive as previously described and per standard clinical practice, with strong positive results (ie, level 3) highly specific for malignancy also examined as previously described.^8^, ^51^, ^55^

### 2.3 Statistical analysis

Demographic differences in cohorts that underwent focused panel testing compared with expanded panel testing were compared by the *Z* test for proportions using the R statistical software (http://r-project.org). The percent differences in the frequency of patients with strong and weak driver mutations or the lack thereof between the cohorts that underwent focused compared with expanded mutation panel testing were performed using the *Z* test for proportions using the R statistical software. These differences were also examined in the subset of patients who had detectable oncogenic change. The *P* values of <.05 were considered statistically significant.

## 3 RESULTS

Molecular results of consecutive patients who underwent clinically prescribed focused mutation panel testing from January 2017 to June 2018 or expanded mutation panel testing from June 2018 to November 2018 were examined. Molecular testing was prescribed by 786 institutions, including various independent pathology practices, community hospital pathology departments, large metropolitan medical centers, and tertiary care academic centers in the United States and Canada. The failure rate of molecular testing was on average 4% among all patients tested, including 3.7% for the focused panel and 4.5% for the expanded panel.

**Figure 1:**
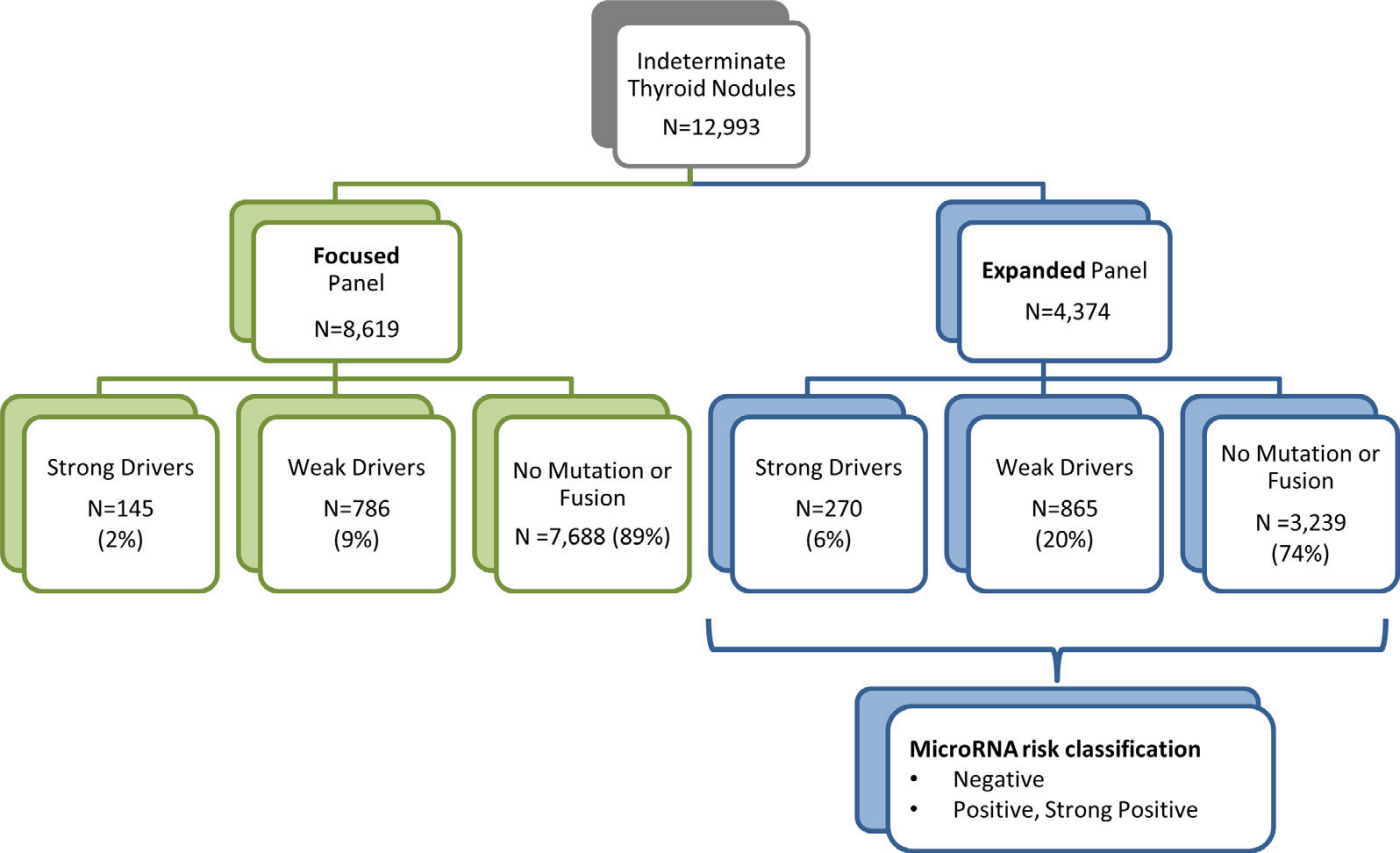
Study cohorts of patients who underwent either focused or expanded mutation panel testing. The number of patients with nodules harboring strong drivers, the number of patients with nodules harboring weak drivers, and the number of patients lacking detectable mutations or fusions are shown

Mutation panel results of 12 993 patients who had assessable molecular results were examined (Figure 1). Patients were divided into two large cohorts. The first underwent focused mutation panel testing (Table 1, gray cells) and the second underwent expanded mutation panel testing (Table 1, gray and white cells). Mutations and fusions were categorized as those strongly predictive of malignancy and aggressive cancer (ie, strong drivers) or those more weakly predictive (ie, weak drivers). *BRAFV600E*, *TERT*, and *RET* mutations and *BRAF*‐ and *RET*‐related fusions were considered strong drivers given their established high PPV for malignancy, *BRAFV600E*‐like signatures, and/or association with aggressive disease, as described in Table 1 (bold font). Other mutations and fusions were considered weak drivers based on the literature supporting their presence in both benign and malignant thyroid nodules, their *RAS*‐like signatures, and/or the lack of literature supporting their high positive predictive value for malignancy or aggressive behavior, as described in Table 1 (regular font).

In total, 8619 patients underwent focused panel testing, while 4374 patients underwent expanded panel testing (Figure 1). Patients who underwent expanded panel testing were also evaluated for the microRNA classification. Patients of both cohorts either had baseline indeterminate cytology with B‐III (AUS/FLUS) or B‐IV (FN/SFN) cytology or did not have a Bethesda diagnostic category available in the data set examined. Given that molecular testing was prescribed as standard of care, it is reasonable to assume that the majority of the latter had either B‐III (AUS/FLUS) or B‐IV (FN/SFN) cytology. This assumption is consistent with our clinical experience, in which only 3% of nodules that have undergone clinically prescribed molecular testing have had indeterminate cytology considered suspicious for malignancy (B‐V). The proportion of females and the proportion of patients with nodules that had B‐IV (FN/SFN) indeterminate cytology did not differ between cohorts (Table 2). The median age of patients only differed by 1 year between cohorts. The expanded panel cohort had more patients with B‐III (AUS/FLUS) cytology, given that more patients in the focused panel cohort did not have a Bethesda diagnostic category available in the data set examined at the time of clinically prescribed molecular testing.

**Table 2:** Demographics of patients who underwent focused and expanded mutation panel testing [Color table can be viewed at http://wileyonlinelibrary.com]

	Focused panel (n = 8619)	Expanded panel (n = 4374)	*P* value
Age median years	58	59	.0143
Female, N (%)	77%	77%	.9678
AUS/FLUS (B‐III) N (%)	5482 (64%)	3294 (75%)	<.0001
FN/SFN (B‐IV) N (%)	1880 (22%)	978 (22%)	.4908
Bethesda Diagnostic Category not available in data set N (%)	1257 (15%)	102 (2%)	<.0001

**Figure 2:**
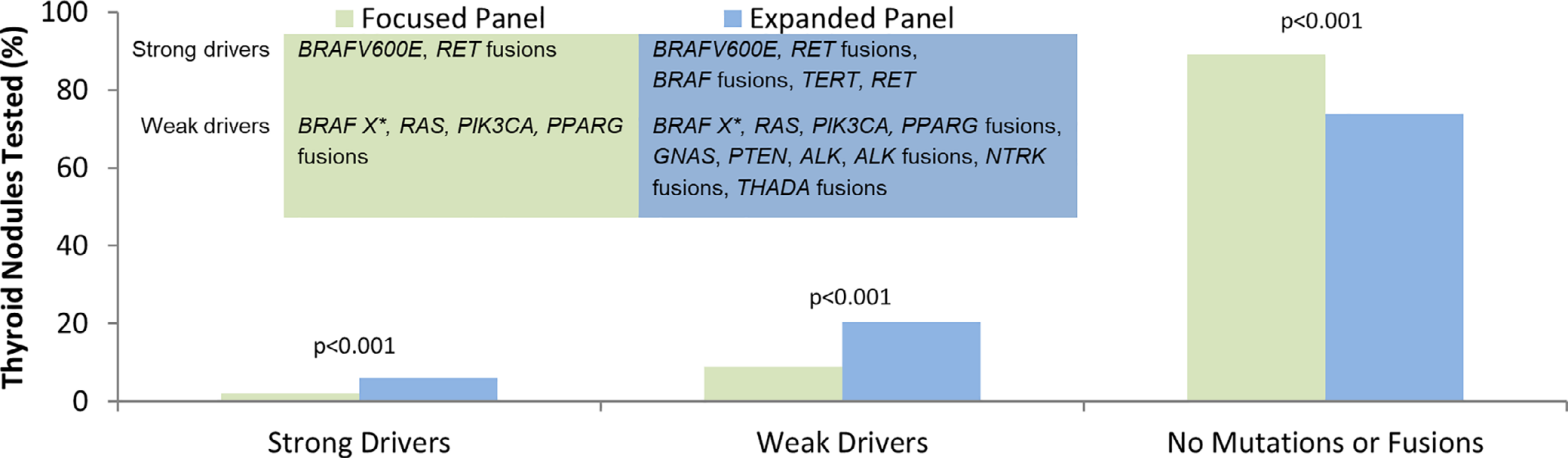
Frequency of patients with strong drivers, weak drivers, or the lack thereof (ie, no mutations or fusions) is shown for patients who underwent focused panel (n = 8619; green) or expanded panel (n = 4374; blue) testing. BRAF X* indicates *BRAF* mutation other than *BRAFV600E*

Most patients (89%) who underwent focused panel testing lacked detectable oncogenic mutations and fusions (Figure 2). This percentage was decreased to 74% in the cohort of patients who underwent expanded panel testing (*P* < .001). Conversely, the percentage of patients who had detectable mutations or fusions was increased in patients who underwent expanded compared with focused panel testing. Most mutations and fusions detected were weak drivers in both panels. Weak drivers were more frequent in patients who underwent expanded (20%) compared with focused (9%) panel testing (*P* < .001) (Figure 2). Strong drivers were also more frequent in patients who underwent expanded (6%) compared with focused (2%) panel testing (*P* < .001) (Figure 2).

**Figure 3:**
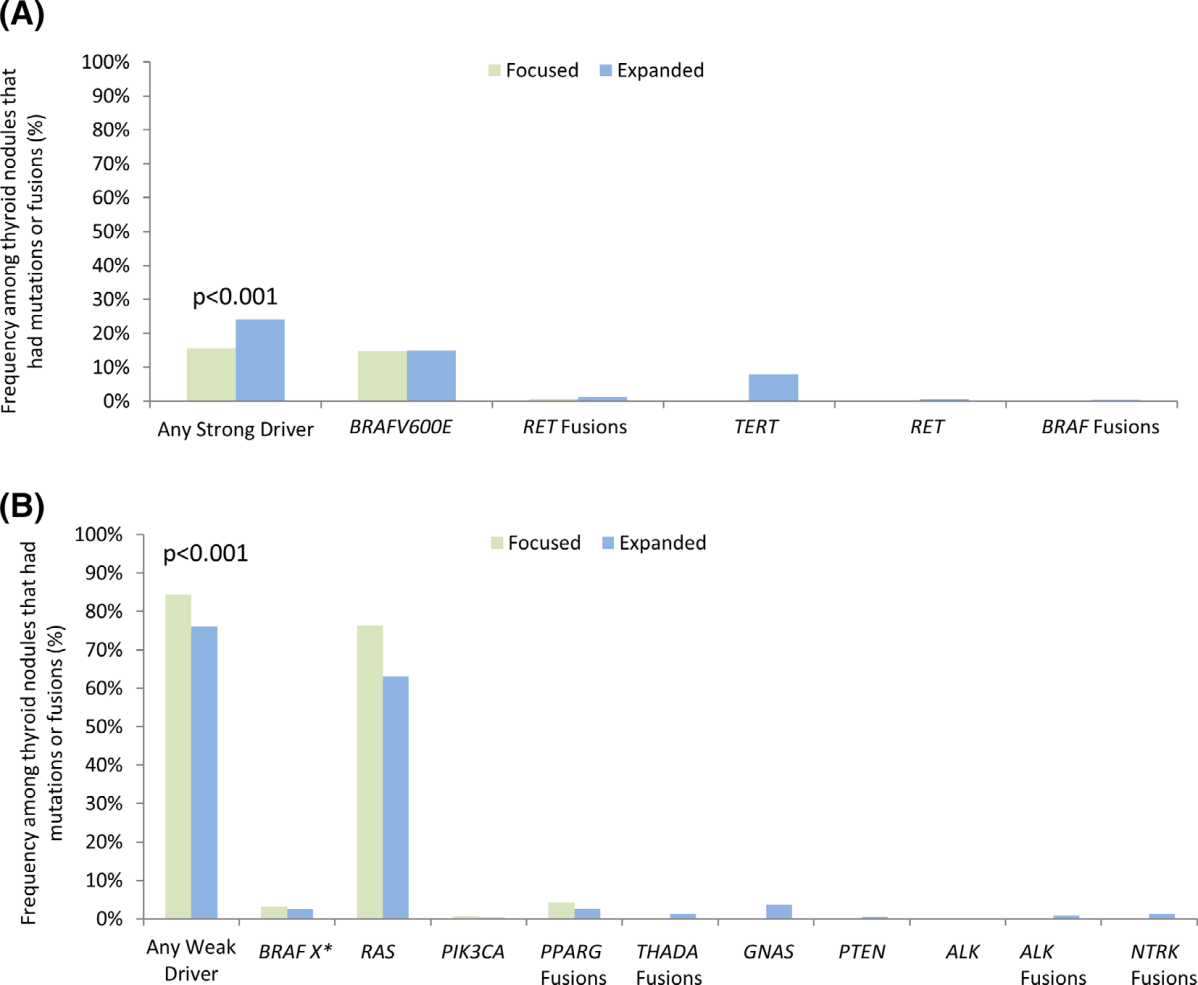
Frequency of (A) strong driver and (B) weak driver mutations and fusions among all patients who had nodules with mutations and fusions detected by the focused (n = 931, green bar) or the expanded (n = 1135, blue bar) mutation panels. BRAF X* indicates *BRAF* mutation other than *BRAFV600E*

Expansion of the mutation panel increased the frequency of patients with strong drivers among patients who had detectable oncogenic change. In these patients, 16% of those who underwent focused panel testing had strong drivers; while 24% of those who underwent expanded panel testing had strong drivers (*P* < .001) (Figure 3A). *BRAFV600E* mutations occurred most frequently in both the focused (15%) and expanded (15%) panel cohorts. Although comparatively infrequent, additional strong drivers were detected by the expanded panel, including *RET* related fusion transcripts, *RET* mutations, *BRAF* related fusions, and *TERT* promoter mutations, with *TERT* being the most common (8%).

Expansion of the mutation panel decreased the frequency of patients with weak drivers among patients who had detectable oncogenic change. In these patients, 84% of those who underwent focused panel testing had weak drivers; while only 76% of those who underwent expanded panel testing had weak drivers (*P* < .001) due to a shift toward detection of strong drivers (Figure 3B). *RAS* mutations were the most frequently detected weak driver in both the focused (77%) and expanded (63%) panel cohorts. Although comparatively infrequent, additional weak drivers were detected by the expanded panel, including *ALK‐*, *NTRK‐*, and *THADA*‐related fusion transcripts and mutations in *PTEN* and *GNAS*, with *GNAS* being the most common (4%). Although *ALK*‐related fusions were detected, no *ALK* mutations were identified.

The coexistence of multiple mutations and fusions occurred infrequently in thyroid nodules of patients who had oncogenic change (Table 3). Expansion of the panel increased the detection of coexisting drivers from occurring in 1% of patients in the focused panel to 5% of patients in the expanded panel (*P* < .001). *TERT* promoter, *BRAFV600E*, *RAS*, *PIK3CA*, and *PTEN* mutations were the most frequent partners of coexisting mutations, with *TERT* and *PTEN* being unique to the expanded panel. Most coexisting drivers were weak drivers found paired with strong drivers, with the majority of those strong drivers detected by the expanded panel. Coexisting *TERT* and *RAS* mutations and coexisting *TERT* and *BRAFV600E* mutations were the most frequent in the nodules of patients who underwent expanded panel testing, occurring in 3% and 1% of patients, respectively. There were no fusions that coexisted with other fusions. A *RAS* mutation coexisted with a *PPARG* fusion in one instance.

**Table 3:** Frequency of patients with nodules harboring multiple coexisting strong and/or weak drivers among patients with nodules harboring mutations and fusions that underwent focused (N = 931, green) or expanded (N = 1152, blue) mutation panel testing [Color table can be viewed at http://wileyonlinelibrary.com]

	Focused panel N = 931 (N%)	Expanded panel N = 1135 (N%)
Coexisting strong drivers		
*BRAF(V600E), TERT*	NA	1.1% (12)
Coexisting strong and weak drivers		
BRAFV600E, BRAF X*	0.1% (1)	0%
*BRAFV600E, RAS*	0.5% (5)	0%
*BRAFV600E, PIK3CA*	0%	0.1% (1)
*TERT, PIK3CA*	NA	0.1% (1)
*TERT, RAS*	NA	3.2% (36)
TERT, BRAF X*	NA	0.1% (1)
*RET, RAS*	NA	0.1% (1)
Coexisting weak drivers		
*RAS, PPARG* fusion	0.1% (1)	0%
*PIK3CA, RAS*	0.2% (2)	0.2% (2)
*PTEN, RAS*	NA	0.1% (1)
*PTEN, GNAS*	NA	0.2% (2)
Total coexisting drivers	1% (9)	5% (57)
*P* value	*P* < .001	

*Notes*: NA, not applicable as both mutations were not tested for in focused panel; BRAF X*, mutations in *BRAF* other than *BRAFV600E*.

**Figure 4:**
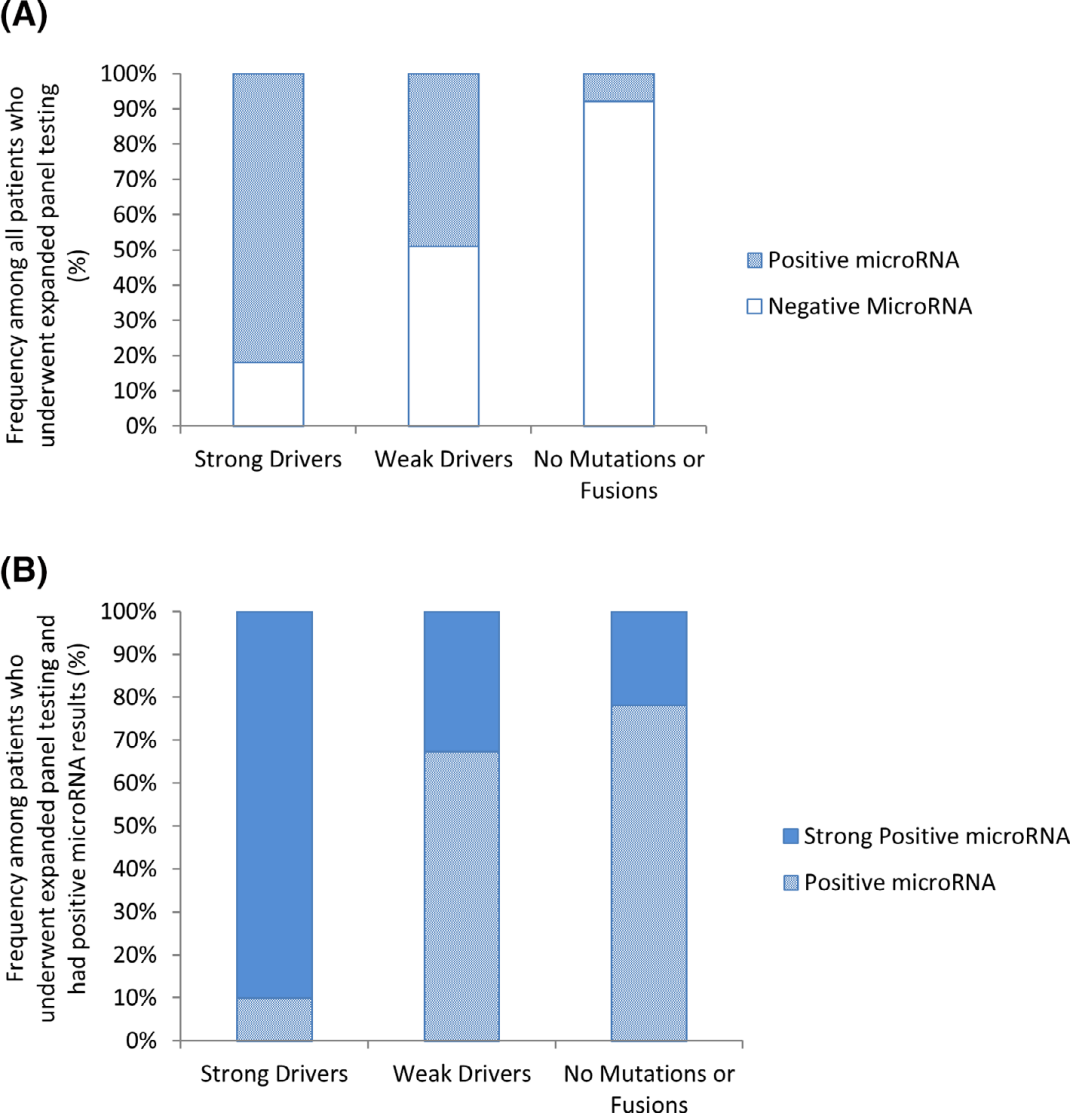
The frequency of (A) positive and negative microRNA classification in all patients who underwent expanded panel testing who had strong drivers (n = 270), weak drivers (n = 865), or the lack thereof (n = 3239) and the frequency of (B) positive and strong positive microRNA classification among all patients who had positive microRNA results based on the presence of strong drivers (n = 222), weak drivers (n = 428), or the lack thereof (ie, no mutations or fusions, n = 246) as determined by expanded mutation panel testing

We examined microRNA results of patients who underwent expanded panel testing to determine if strong drivers, weak drivers, or the absence of both were associated with previously described positive or negative microRNA risk classifications that have high (85%) specificity for malignancy when used in combination with mutational analysis.^51^ Among patients who had positive microRNA results, we also further examined the frequency of strong positive microRNA results, as this level of microRNA has been shown to have even higher specificity for malignancy.^8^ Positive microRNA results were observed in 82% of patients with strong drivers (Figure 4A). The majority of these patients (90%) had strong positive microRNA results (Figure 4B). Approximately half of patients (49%) with weak drivers had positive microRNA results (Figure 4A) of which 33% had strong positive microRNA results (Figure 4B). Of patients who lacked both strong and weak drivers, only 8% had positive microRNA results (Figure 4A), 22% of which were strong positive (Figure 4B).

## 4 DISCUSSION

Some mutations and fusions included in mutation panels are highly predictive of thyroid malignancy and aggressive cancer and as such can be considered strong drivers of malignancy (ie, strong drivers). Many other mutations and fusions, such as commonly detected *RAS* mutations, can occur in both benign and malignant thyroid nodules, have *RAS*‐like signatures, and/or lack support for their PPV for malignancy,^8^, ^14^, ^22^, ^23^, ^24^, ^25^, ^26^, ^27^, ^28^, ^29^, ^30^, ^31^, ^32^, ^33^, ^34^, ^35^, ^36^, ^37^, ^38^, ^39^, ^40^, ^41^, ^42^ and as such can be considered more weakly associated with malignancy (ie, weak drivers). We examined strong and weak driver mutations and fusions in thyroid nodules that underwent clinically prescribed molecular testing with either focused or expanded mutation panels to better understand the incremental utility in using an expanded mutation panel and how microRNA classifier testing can provide additional diagnostic information to expanded panel test results.

Expansion of the panel increased detection of strong drivers by 8% in patients who had oncogenic change. The higher frequency of strong drivers in patients who underwent expanded panel testing was largely due to inclusion of *TERT* promoter mutations in the expanded panel. *TERT* promoter mutations have been associated with poorly differentiated and anaplastic thyroid cancer.^16^, ^17^, ^18^, ^19^, ^21^ They are also considered an independent risk factor for persistent disease, distant metastases, and mortality for well differentiated thyroid cancer.^16^, ^20^ Given this prognostic information, inclusion of *TERT* promoter mutation testing in the expanded panel may provide enhanced clinical utility. In our study, nodules with strong drivers, such as *TERT*, typically had positive microRNA results consistent with high risk of malignancy, the majority of which were strong positive microRNA results that are highly specific for malignancy.^8^ While the positive predictive value of *TERT* and other strong drivers is not always 100%, their high correlations to positive and strong positive microRNA results support their strong association high risk of malignancy.

Inclusion of less common mutations and fusions in the expanded panel increased the detection of multiple coexisting mutations by 4%, which further increased utility in identifying aggressive forms of thyroid cancer. Many of these would have been otherwise missed using a more focused panel. The majority of coexisting drivers were weak drivers paired with strong drivers unique to the expanded panel. Coexistence of weak and strong drivers not only elevates concern for malignancy but also elevates concern for aggressive thyroid cancer. Coexisting *TERT* promoter mutation with *BRAFV600E* or *RAS* mutation was the most common, with such coexisting mutations having the potential to promote aggressive tumor behavior and predict poor patient survival.^17^, ^19^, ^43^, ^45^ It is well established that poorly differentiated and anaplastic thyroid cancer harbors multiple oncogenic drivers, including coexisting RAS and PIK3CA mutations detected in our study.^21^ Well‐differentiated papillary cancers can also have multiple oncogenic drivers, which typically indicate aggressive tumor behavior.^28^, ^29^, ^33^, ^44^ In such cases, these molecular findings may enable an optimal surgical approach to include lymph node sampling.

All oncogenic changes can contribute to neoplastic growth and progression, and therefore both strong and weak drivers should be considered clinically important. However, the individual detection of drivers that are more weakly associated with malignancy presents a challenge to guiding patient management.^25^ Expansion of the panel increased the overall frequency with which weak drivers were found, but decreased the frequency of weak drivers in patients who had oncogenic change by 8% due to a shift toward detection of strong drivers. Not surprisingly, weak driver *RAS* mutations were the most common drivers detected, occurring in 63% of patients with oncogenic change in the expanded panel. Malignancy risk can range from approximately 15% to 70% in indeterminate nodules with *RAS* mutations leaving uncertainty as to the presence of cancer.^8^, ^25^ Our results support that subsequent microRNA testing can help to overcome this uncertainty. Approximately half of nodules with weak drivers had positive microRNA results consistent with a higher risk of malignancy.^51^ A significant portion (33%) of those with positive microRNA results had strong positive microRNA levels that are highly specific for malignancy and that are prevalent in nodules with strong drivers, as we have demonstrated herein. 8 Positive microRNA results and even more so strong positive results favor elevated concern for malignancy.^8^ Consistently, results of a large multicenter study examining the outcomes of patients who underwent clinically prescribed combination mutation and microRNA testing concluded that more aggressive surgical management options are warranted in patients with weak drivers and positive microRNA results given confirmed high risk of malignancy.^52^

A small, but nonetheless significant, minority of thyroid follicular cancers will lack detectable mutations and fusions.^7^, ^8^, ^9^ Thus, their absence cannot fully exclude malignancy. Although expansion of the panel reduced the frequency, the majority of patients who underwent focused and expanded panel testing lacked detectable mutations and fusions. There are two fundamental mechanisms that can account for lack of detectable mutations and fusions in malignant nodules. Despite examining a broad panel of oncogenic drivers, such as in the expanded panel examined herein, a subset of thyroid cancer may be driven by rare, as of yet uncharacterized oncogenic changes. Alternatively, the distribution of cells harboring an oncogenic change within a given neoplasm may be heterogeneous such that the needle aspirate secures a subset of cells that do not harbor the oncogenic change. With respect to either mechanism, the lack of detectable oncogenic change cannot provide complete assurance that a nodule is benign and as such those nodules have a residual cancer risk of 5% to 25%.^7^, ^8^, ^9^

Our results support that microRNA testing can help to overcome limitations in assessing malignancy risk in the absence of detectable mutations and fusions. The extracellular distribution of microRNAs may help to identify higher risk lesions even when mutational heterogeneity and sampling variability may be present and no mutations or fusions are detectable.^51^ Our results demonstrate that strong positive microRNA results that are highly specific for malignancy^8^ and prevalent in nodules with strong drivers can also be found in a subset of patients who lack any detectable mutations and fusions even when a more expanded mutation panel is used. Additional molecular testing that is highly specific for malignancy can help to identify patients at higher risk of malignancy in this subgroup. Given the reported high specificity of positive microRNA results and their strong association with strong driver oncogenic changes predictive of malignancy demonstrated here, positive and even more so strong positive microRNA results can support elevated concern for malignancy and can justify more aggressive treatment options even in the absence of detectable mutations and fusions.^8^, ^51^ This is further supported by results of a multicenter study for the outcomes of patients who underwent clinically prescribed combination mutation and microRNA testing concluding that more aggressive management options are warranted in patients that lack mutations and fusions but have positive microRNA results given confirmed high risk of malignancy.^52^

Although combination mutation and microRNA testing has been validated in patients with surgically confirmed outcomes,^51^ our study was limited to examining the frequency of those results in a very large cohort of patients who underwent clinically prescribed molecular testing, where outcomes of patients were unknown and where an expanded mutation panel was used in combination with microRNA testing. However, the utility of this combination testing described herein is consistent with that demonstrated in a multicenter study that examined the outcomes of patients who underwent clinically prescribed combination mutation and microRNA testing.^52^ In this study, positive microRNA results identified high risk of cancer in patients who had weak driver oncogenic changes and in those who lacked oncogenic change, concluding that more aggressive surgical management options were justified based on positive microRNA results given high risk of cancer confirmed in the study. Our study is the first to examine this approach in a large cohort of patients to better understand the frequency with which various molecular scenarios can be encountered (ie, strong drivers, weak drivers, no oncogenic change) and to better understand the utility of using the combination of an expanded mutation panel with microRNA risk classification.

Understanding the nuanced differences between specific forms of oncogenic damage can provide significant advancements in the diagnosis and treatment of thyroid cancer. Using expanded mutation panels that include less common mutations and fusions can offer increased utility when used in combination with microRNA risk classification. Use of additional microRNA analysis to further stratify malignancy risk may help with challenging management decisions encountered in the majority of thyroid nodules with indeterminate cytology, where oncogenic changes that are more weakly predictive of malignancy are found or when no oncogenic changes are detected.

## CONFLICT OF INTEREST

S.D.F., G.K., N.T., S.J., and C.M.N. are employees of Interpace Diagnostics. The remaining authors have no conflicts to declare.

## AUTHOR CONTRIBUTIONS

S.D.F., G.K., N.T., S.J., C.M.N., A.B.B., and J.F.S. each participated in generating data for the study, reviewed data analysis and manuscript drafts, and approved the final draft of the manuscript.
